# Development and clinical application of loop-mediated isothermal amplification combined with lateral flow assay for rapid diagnosis of SARS-CoV-2

**DOI:** 10.1186/s12879-023-08924-3

**Published:** 2024-01-15

**Authors:** Jin Tang, Jie Zhu, Jie Wang, Haiyong Qian, Zengxin Liu, Ru Wang, Qingqing Cai, Yuan Fang, Weifeng Huang

**Affiliations:** 1 Department of Clinical Laboratory, Shanghai Sixth People’s Hospital Affiliated to Shanghai Jiao Tong University School of Medicine, Shanghai, 200233 China; 2 Shanghai Sixth People’s Hospital Affiliated to Shanghai Jiao Tong University School of Medicine, Shanghai, 200233 China; 3 Shanghai Jiao Tong University affiliated the Eighth People’s Hospital, Shanghai, 200235 China; 4 Shanghai Fengxian District Central Hospital, Shanghai, 201406 China; 5 Shanghai Fengxian District Guhua Hospital, Shanghai, 201499 China; 6 Department of Intensive Care Medicine, Shanghai Sixth People’s Hospital Affiliated to Shanghai Jiao Tong University School of Medicine, Shanghai, 200233 China; 7 Genoxor Medical Science and Technology Inc., No 555 Wangfang Road, Minhang District, Shanghai, 201112 China

**Keywords:** SARS-CoV-2, COVID-19, RT-LAMP, Isothermal amplification, Diagnostics, Molecular testing

## Abstract

**Background:**

The diagnostic assay leveraging multiple reverse transcription loop-mediated isothermal amplification (RT-LAMP) could meet the requirements for rapid nucleic acid detection of severe acute respiratory syndrome coronavirus 2 (SARS-CoV-2).

**Methods:**

The devised assay merged the lateral flow assay with the RT-LAMP technology and designed specific primers for the simultaneous detection of the target and human-derived internal reference genes within a single reaction. An inquiry into the assay's limit of detection (LOD), sensitivity, and specificity was carried out. The effectiveness of this assay was validated using 498 clinical specimens.

**Results:**

This LOD of the assay was determined to be 500 copies/mL, and there was no observed cross-reaction with other respiratory pathogens. The detection results derived from clinical specimens showed substantial concordance with those from real-time reverse transcription-polymerase chain reaction (RT-qPCR) (Cohen's kappa, 0.876; 95% CI: 0.833-0.919; *p*<0.005). The diagnostic sensitivity and specificity were 87.1% and 100%, respectively.

**Conclusion:**

The RT-LAMP assay, paired with a straightforward and disposable lateral immunochromatographic strip, achieves visual detection of dual targets for SARS-CoV-2 immediatly. Moreover, the entire procedure abstains from nucleic acids extraction. The samples are lysed at room temperature and subsequently proceed directly to the RT-LAMP reaction, which can be executed within 30 minutes at a constant temperature of 60-65°C. Then, the RT-LAMP amplification products are visualized using colloidal gold test strips.

**Trial registration:**

This study was registered at the Chinese Clinical Trial Registry (Registration number: ChiCTR2200060495, Date of registration 2022-06-03).

**Supplementary Information:**

The online version contains supplementary material available at 10.1186/s12879-023-08924-3.

## Background

The SARS-CoV-2 (severe acute respiratory syndrome Coronavirus 2) is regarded as a highly infectious and pathogenic virus, and has caused a global epidemic of novel coronavirus disease 2019 (COVID-19) and continues to this day [1]. Early diagnosis of SARS-CoV-2 infections is essential for preventing further transmission and afford appropriate treatment. However, diagnosis is challenging due to the non-specific symptoms and radiological characteristics of COVID-19, which resemble the common cold and influenza. Confirmation of SARS-CoV-2 infection depends solely on detecting the presence of viral RNA [2]. The reverse transcription-quantitative polymerase chain reaction (RT-qPCR) is the most widely used in clinical laboratories to detect the novel coronavirus, thus becoming the gold standard of the SARS-CoV-2 infection diagnosis [3, 4]. While RT-qPCR assay exhibits excellent analytical performance, it does present several limitations: lengthy detection times (1-2 hours), being restricted to clinical laboratory conditions, and the requirement for specialized instruments and trained personnel [5, 6]. These constraints render RT-qPCR tests inadequate for the large-scale population screening. Therefore, considering the virus's rapid mutation, there is an urgent requirement exists for a quicker, simpler, and more sensitive methodology to swiftly identify infected patients across varying settings.

Loop-mediated isothermal amplification (LAMP) technology has become an important technical route for the development of rapid nucleic acid detection, which has been applied to detect viruses, bacteria, and other pathogens, due to its high sensitivity, specificity, short reaction time, and minimal laboratory infrastructure requirements [7, 8]. The LAMP method generally uses 6 primers – a combination of 4 primers specific to the target DNA and 2 additional loop primers. RT-LAMP assays allowing for reverse transcription and DNA amplification within just 30 minutes at a steady temperature of 60-65℃. DNA denaturation is unnecessary in the presence of Bst DNA polymerase, and only requires isothermal conditions to form a dumbbell-shaped DNA structure that can serve as a template for further amplification. The unique rapid self-triggered amplification of LAMP reaction could be achieved by adding loop primers that complemented dumbbell-shaped DNA [9, 10]. RT-LAMP assays have been explored for diagnosing SARS-CoV-2 RNA, and through visual turbidimetry or fluorescence real-time detection, this method has become a practical and rapid nucleic acid detection method [11, 12]. However, most of these tests focus on individual target genes, lacking comprehensive quality control over the sampling and testing process, which can lead to unreliable diagnostic results.

Lateral flow assays (LFA), a paper-based platform, enables the detection the analytes in complicated mixtures, displaying the results within 5-30 minutes. A key feature of fluorescent probe-based nucleic acid lateral flow tests is the addition of a detection probe labelled with a modification group to the PCR reaction. As the PCR product is applied to the lateral flow strip, the target and detection probe migrate through the membrane via capillary action, forming a sandwich-like hybridisation product with antibodies specific to the modification group that can bind to the PCR product when immobilised in advance on a nitrocellulose membrane. During the assay, different target primers are labelled with biotin, fluorescein isothiocyanate (FITC), or 6-carboxyfluorescein (6-FAM), and both markers are integrated onto the double-stranded amplification product post-amplification. The flexibility of the method enables capture probes to be immobilised at various positions on the test strip, and fluorophore-labelled probes can be labelled with different fluorophores. This flexibility permits the hybridization of many different single-stranded DNA products, generated in multiplex PCR, for detection in single lateral flow assays. To this end, we developed and validated a novel RT-LAMP assay to detect novel coronaviruses. This assay introduces the detection of human endogenous quality control targets alongside the coronavirus targets, and uses a lateral flow immunochromatographic strip chromogenic technique to interpret the results, with excellent sensitivity from the heated colloidal gold particles (AuNP) coupled to a binding probe, ensuring reliable results.

For nucleic acid detection of SARS-CoV-2, it’s crucial to unify all steps into a streamlined workflow for the development of a point-of-care nucleic acid detection method. This will shorten the operating time and procedures. Firstly, the nucleic acid extraction step should be eliminated. Secondly, the reliance on precision instruments and equipment must be educed. Lastly, amplicons are usually analyzed via fluorescence signals, thus requiring specialized instruments and additional operational steps.

In this study, we developed a rapid nucleic acid method for SARS-CoV-2. This method combined a one-step reverse transcription and LAMP detection method, then chose a simple and disposable lateral flow immunochromatographic strip for the immediate interpretation of the amplified coronavirus nucleic acid test results. The diagnosis of SARS-CoV-2 in a clinical sample can be achieved in less than 40 minutes, from swab sample to diagnostic result. This makes it a significant development direction for rapid nucleic acid diagnosis of COVID-19 and enables large-scale population screening for the novel coronavirus, particularly in resource-limited settings.

## Methods

### RT-LAMP primer design

The RT-LAMP primers were designed based on the genomic sequences of SARS-CoV-2 (as announced in NCBI GenBank, accession: NC_045512, location 28, 274-29, 533), directed reference to the conserved sequence region of the novel coronavirus N gene, as disclosed by the Chinese Center for Disease Control. The primer design software used was Primer Explorer V4 (Eiken Chemical Co. LTD, Tokyo, Japan). The primer set includes two external primers (forward primer F3 and reverse primer B3), two internal primers (forward primer FIP and reverse primer BIP) and two loop primers (forward primer LF and reverse primer LB). The primers’ specificity was validated through a BLAST prior to synthesis. The 5’ end of one accelerated primer was labeled with fluorescein isothiocyanate (FITC) and tetramethylrhodamine (TAMRA), while another primer’s 5’ end was labeled using biotin and digoxin. This ensured imultaneous incorporation of both labels into the double-stranded amplification product, specifically bound to the colloidal gold-labeled antibody and the streptavidin immobilized on the test strip. All primers were synthesized by Suzhou Synbio Technologies (China). The primer sequences are provided in Table 1.

**Table 1:** Primer sequences of RT-LAMP detection

**Primer**	**Sequence (5’- 3’)**
ACTB-F	AGTACCCCATCGAGCACG
ACTB-B	AGCCTGGATAGCAACGTACA
ACTB-FIP	GAGCCACACGCAGCTCATTGTATCACCAACTGGGACGACA
ACTB-BIP	CTGAACCCCAAGGCCAACCGGCTGGGGTGTTGAAGGTC
ACTB-LF	Digoxigenin--TGTGGTGCCAGATTTTCTCCA
ACTB-LB	TAMRA--CGAGAAGATGACCCAGATCATGT
N2-F	ACCAGGAACTAATCAGACAAG
N2-B	GACTTGATCTTTGAAATTTGGATCT
N2-FIP	TTCCGAAGAACGCTGAAGCGGAACTGATTACAAACATTGGCC
N2-BIP	CGCATTGGCATGGAAGTCACAATTTGATGGCACCTGTGTA
N2-LF	Biotin--GGGGGCAAATTGTGCAATTTG
N2-LB	FITC--CTTCGGGAACGTGGTTGACC

### RT-LAMP assay

The multiple RT-LAMP assays were conducted with a reaction volume of 50 µL, containing lysate (the lysis buffer comprised 200mM Tris-HCL, 500mM KCL, 100mM (NH_4_)_2_SO_4_, 80mM MgSO_4_ and 1% Triton-100) of 25 µL, 5× reaction buffer of 5 µL, a RT-LAMP primer mixture (FIP, 16 µM; BIP, 16 µM; F3, 2 µM; B3, 2 µM; LF, 4 µM; LB, 4 µM) of 5 µL, reverse transcriptase (Hifair® III Reverse Transcriptase, Yeasen Biotechnology (Shanghai) Co. Ltd., China) of 0.5 µL, Bst enzyme (Hieff® Bst Plus DNA Polymerase, Yeasen Biotechnology(Shanghai) Co., Ltd., China) of 0.5 µL, 50× LAMP fluorescent dye (New England Biolabs, UK) of 1 µL, and ultrapure water of 13 µL. The SARS-COV-2-N pseudovirus samples used in this study were commercially obtained from Tsingke Biotechnology Co. Ltd., China. The RT-LAMP reaction was performed in a SLAN-96S fluorescent PCR instrument (Shanghai Hongshi Medical Technology Inc., China) at 60℃ for 30 minutes to collect FAM channel fluorescence. Reaction products were developed for colour using double strip colloidal gold test strips (Beijing Baoying Tonghui Biotechnology Co. Ltd., China). The non-template control (NTC) was set up in each experiment to ensure the absence of contamination. Each experiments in this study was conducted with two parallel biological replicates, and the results obtained were consistent.

**Fig. 1:**
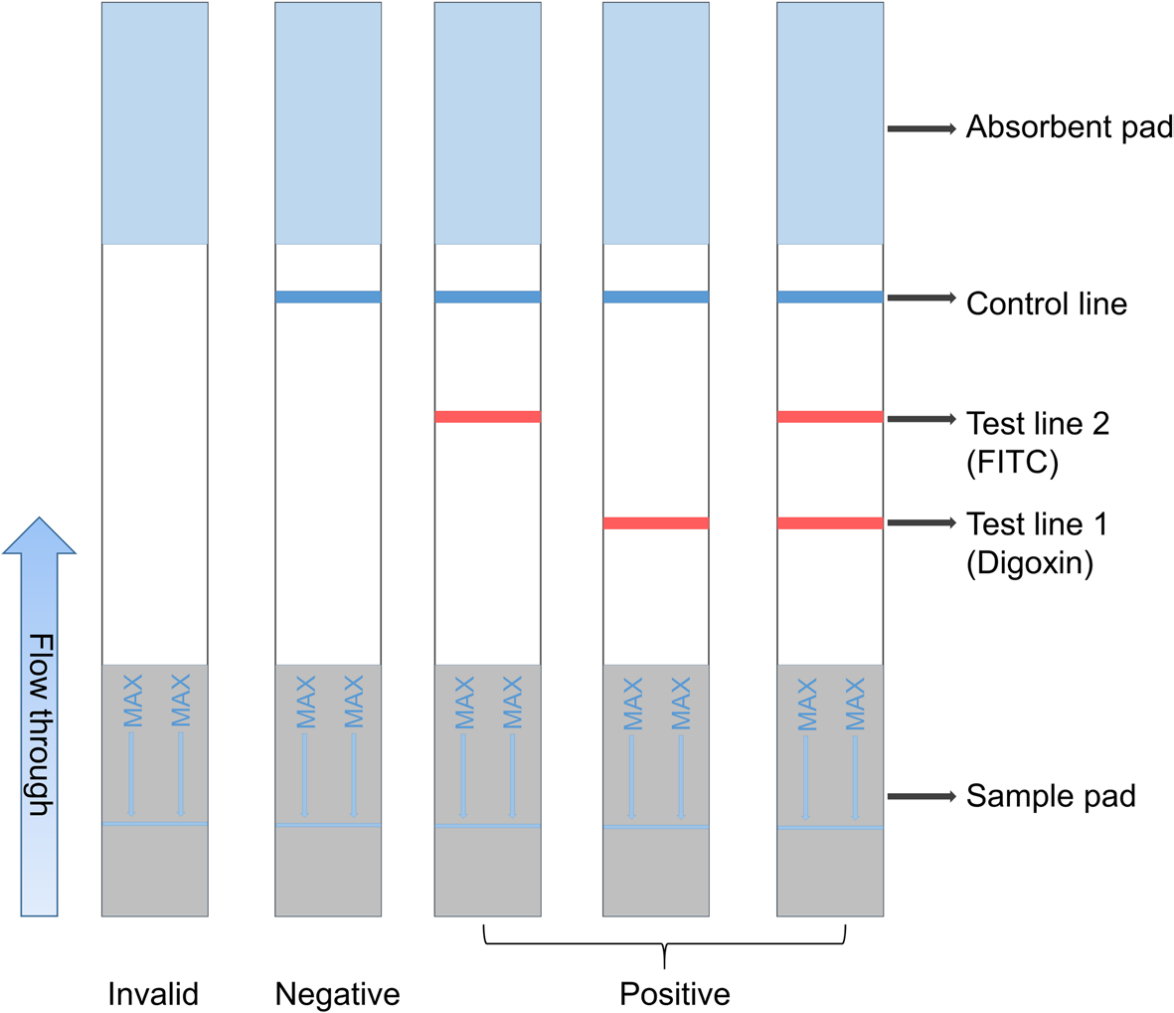
The plane structure diagram of the lateral flow strip. The lateral flow strip includes a sample pad (the zone used to load the LAMP product), a detection zone consisting of test line 1, test line 2, and control line, respectively immobilized with anti-Digoxin, anti- FITC, and biotin, as well as an absorbent pad. Flow through indicates the flow direction of the sample on the strip. The MAX lines indicate the position where the sample is loaded on the sample pad

20 µL of the amplification product was added to 180 µL of ultrapure water for dilution, and then inserted into the test strip for chromogenic reaction. The strip was then interpreted after standing for 5 minutes. The novel coronavirus N gene target and the human internal reference ACTB gene were tested simultaneously in this study. If both target were chromogenic, the test strip indicated a positive result; whereas, if only the ACTB gene target displayed chromogenicity, the test strip was interpreted as negative. In addition, the presence of only the control line (C line) with color rendered a negative result. However, if no C line was displayed, it suggested potential damage to the test strip rendering the result invalid. Figure 1 illustrates the structure of the lateral flow strip.

### RT-LAMP performance evaluation

To determine the lower limit of detection for the RT-LAMP assay, a gradient dilution series of samples with pseudoviruses of the coronavirus N gene was used as template in the RT-LAMP reaction. The extent of dilution yielding the least concentration that still allowed for a positive reaction was recorded. For cross-activity testing, eight common viral pathogens were used including influenza A virus (FluA), influenza B virus (FluB), human coronavirus 229E (HCoV-229E), human parainfluenza virus (HPIV), human rhinovirus (HRV), human metapenumovirus (HMPV), enterovirus (EV), and human cytomegalovirus (HCMV). The samples used were derived from nucleic acid samples that showed positive results in clinical mNGS tests. Finally, clinical samples were utilized to determine the sensitivity, specificity, positive predictive value (PPV) and negative predictive value (NPV) of RT-LAMP assay, for assessing its diagnostic accuracy compared to the gold standard RT-qPCR assay.

### Clinical specimens

This study was approved by the ethical committee of Shanghai Sixth People’s Hospital. From May 1^st^ to June 20^th^ 2021, the nasopharyngeal swabs were clinically collected from 498 COVID-19 patients and a control population at the Shanghai Sixth People's Hospital. In accordance with the diagnostic criteria for SARS-CoV-2 infection published by the Chinese CDC (Center for Disease Control and Prevention), the diagnosis is based on the presence of chest imaging features consistent with viral pneumonia and positive RT-qPCR test results. Each collector underwent a standard set of COVID-19 investigations to test for SARS-CoV-2 infection.

### RNA extraction and real-time fluorescent PCR assay

A commercial RNA extraction kit (Guangzhou Da’an Gene Co. Ltd., China) was used to extract RNA. After initial testing, the samples were aliquoted and preserved. Every single sample of RNA was tested by RT-qPCR in a Biosafety Level 2 Laboratory, utilizing the 2019-nCoV Nucleic Acid Detection Kit (Fluorescent PCR Method) (Guangzhou Da’an Gene Co. Ltd., China). Follow the product instructions, the RT-qPCR detection result was determined based on the CT value: if there was no amplification curve for FAM and VIC channels, or if the CT value was >30, and the CT value of CY5 channel was <30, it was determined as negative; FAM or VIC channels had amplification curves, and the CT value was ≤ 30, it was considered positive.

### Statistical analysis

The results of RT-LAMP assays derived from clinical samples were comprehensively analyzed via SPSS 20.0. In evaluate the reliability of the three RT-qPCR methods, Cohen’s Kappa coefficient was used. Concurrently, the overall diagnostic accuracy, which encompassed sensitivity, specificity, the positive predictive value and the negative predictive value were calculated. All statistical methods were considered significant at a confidence level of 0.05.

## Results

### RT-LAMP primer screen

**Fig. 2:**
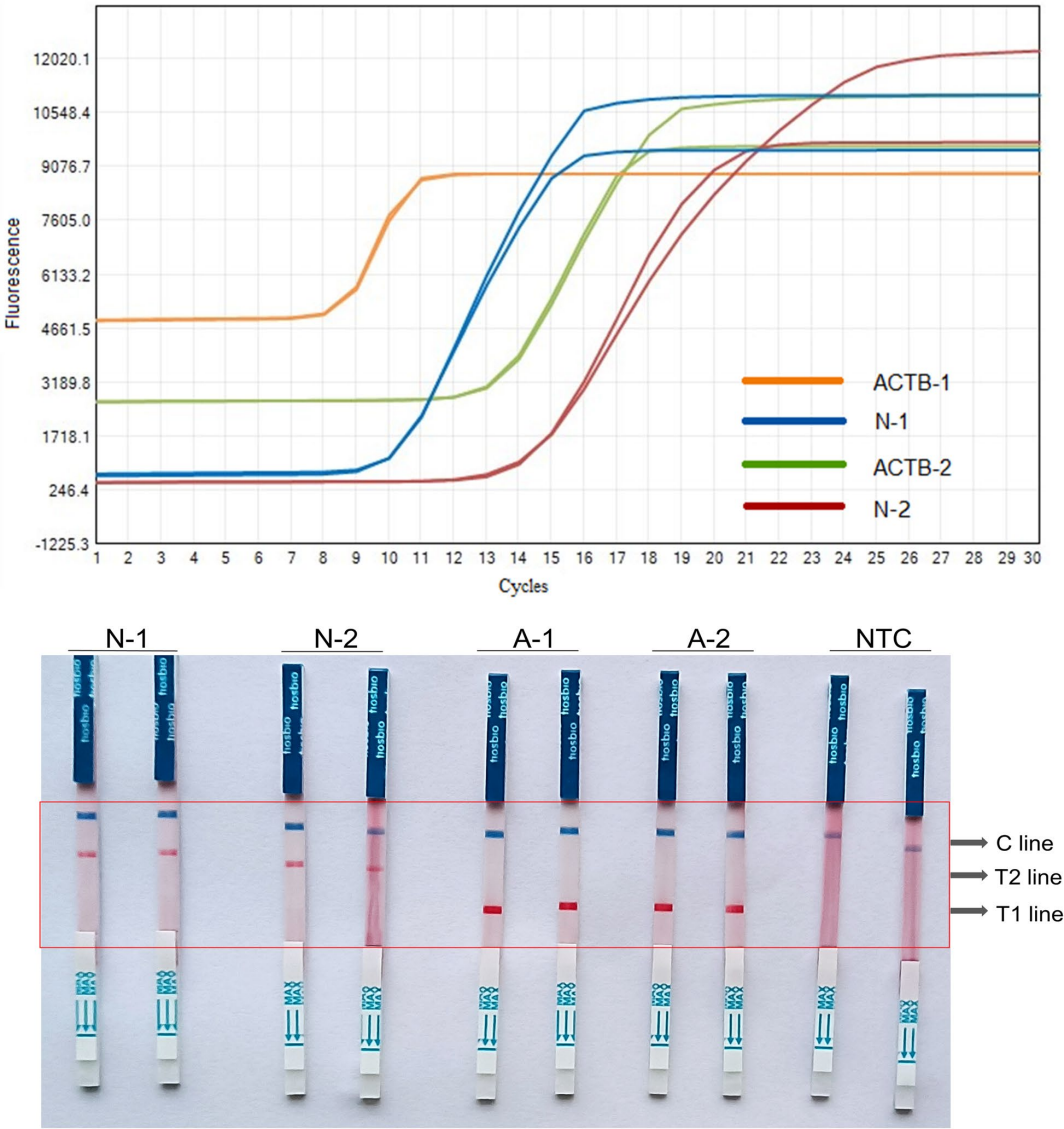
The RT-LAMP results of single target primer. The fluorescence amplification curves were shown on the top, and the coloration reaction results were shown on the bottom. N-1, novel coronavirus N gene primer 1; N-2, novel coronavirus N gene primer 2; ACTB-1 (A-1), internal reference gene human-derived ACTB gene primer 1; ACTB-2 (A-2), internal reference gene human-derived ACTB gene primer 2. NTC, negative control. T1 line, Test line 1 (ACTB gene); T1 line, Test line 2 (N gene); C line, Control line

**Fig. 3:**
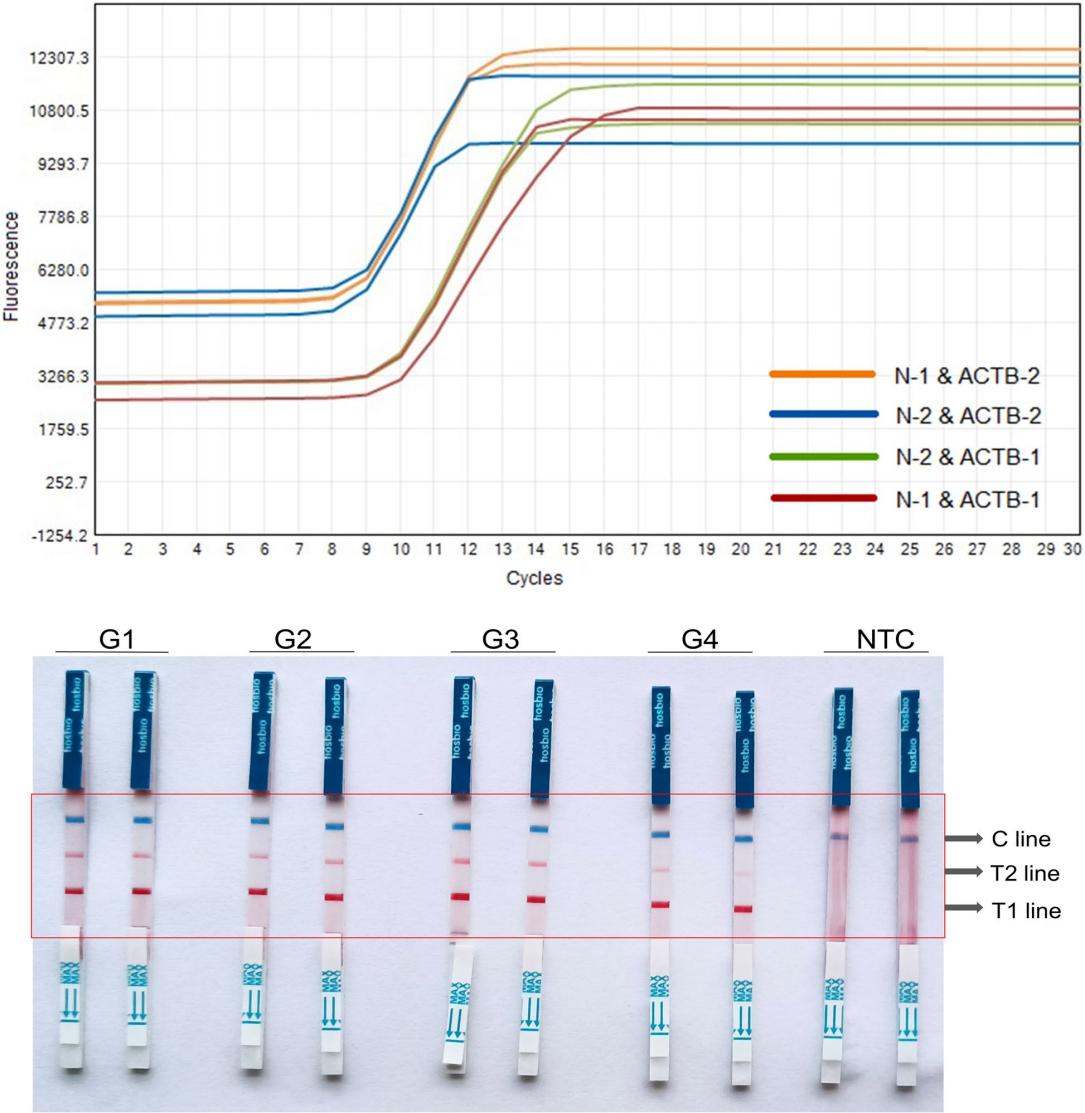
The RT-LAMP results of dual target primer. The fluorescence amplification curves were shown on the top, and the coloration reaction results were shown on the bottom. G1, N-1 and ACTB-1; G2, N-2 and ACTB-1; G3, N-1 and ACTB-2; G4, N-2 and ACTB-2. NTC, negative control. T1 line, Test line 1 (ACTB gene); T1 line, Test line 2 (N gene); C line, Control line

To achieve a RT-LAMP primer combination with superior specificity and enhanced amplification efficiency, we performed nucleic acid amplification using two distinct primer pairs. We found that the N-1 primer designed for the novel coronavirus N gene amplified more efficiently than the N-2 primer, generating CT values of 10 and 14, respectively. Among the human-derived ACTB internal reference primers we designed, the ACTB-1 primer amplified more efficiently than the ACTB-2 primer, with CT values of 9 and 13, respectively (Fig. 2). Subsequently, we mixed the N gene primer set and the ACTB gene primer set for testing. Based on the amplification efficiency and coloration results (Fig. 3), we selected the N-1 gene primer from the N gene primers set and the ACTB-2 gene primer from human ACTB gene primers set as the final combination in this study.

### Optimization of RT-LAMP reaction conditions

**Fig. 4:**
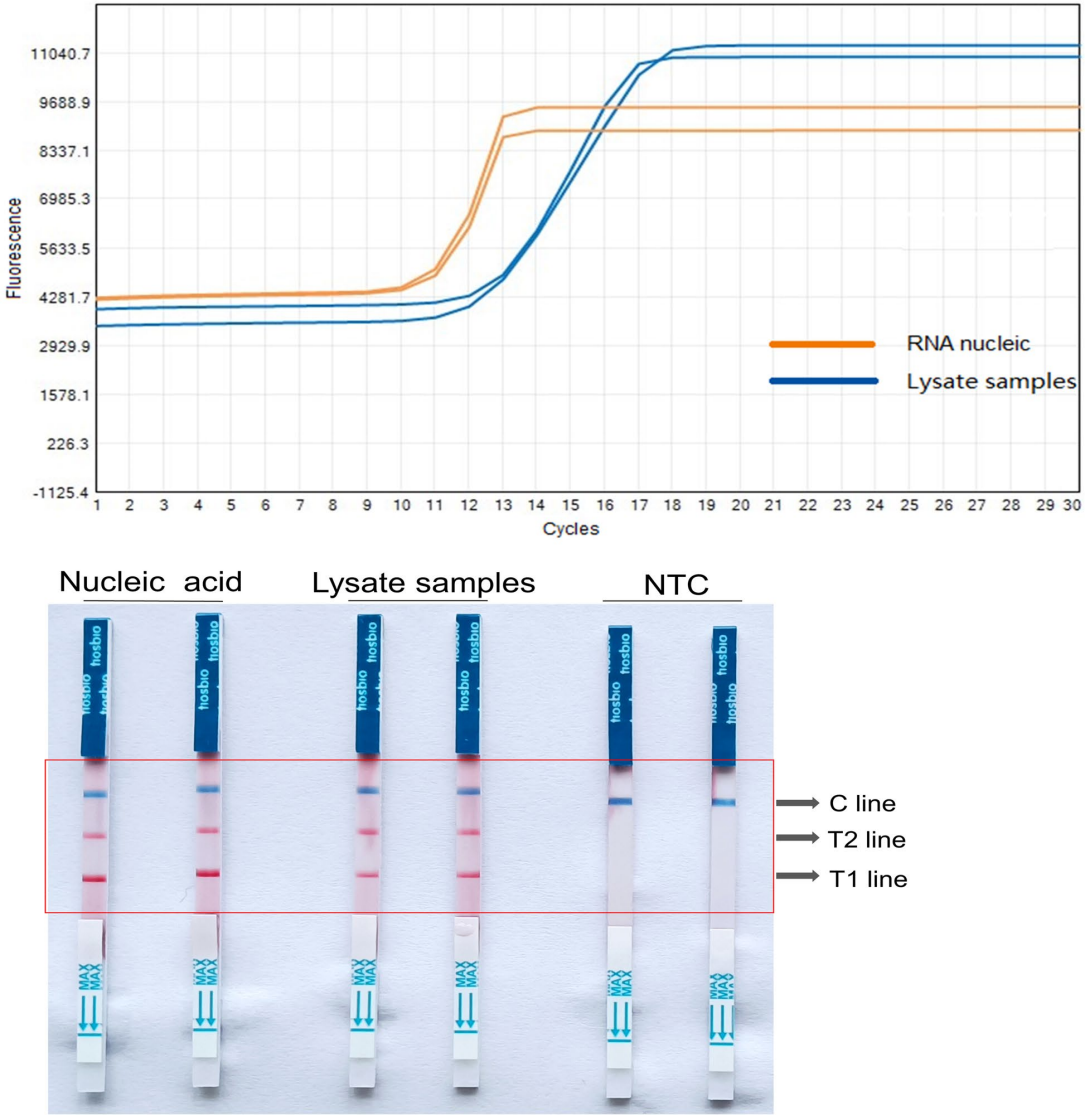
The RT-LAMP results of lysate samples and nucleic acid samples. The fluorescence amplification curves were shown on the top, and the coloration reaction results were shown on the bottom. NTC, negative control. T1 line, Test line 1 (ACTB gene); T1 line, Test line 2 (N gene); C line, Control line

**Fig. 5:**
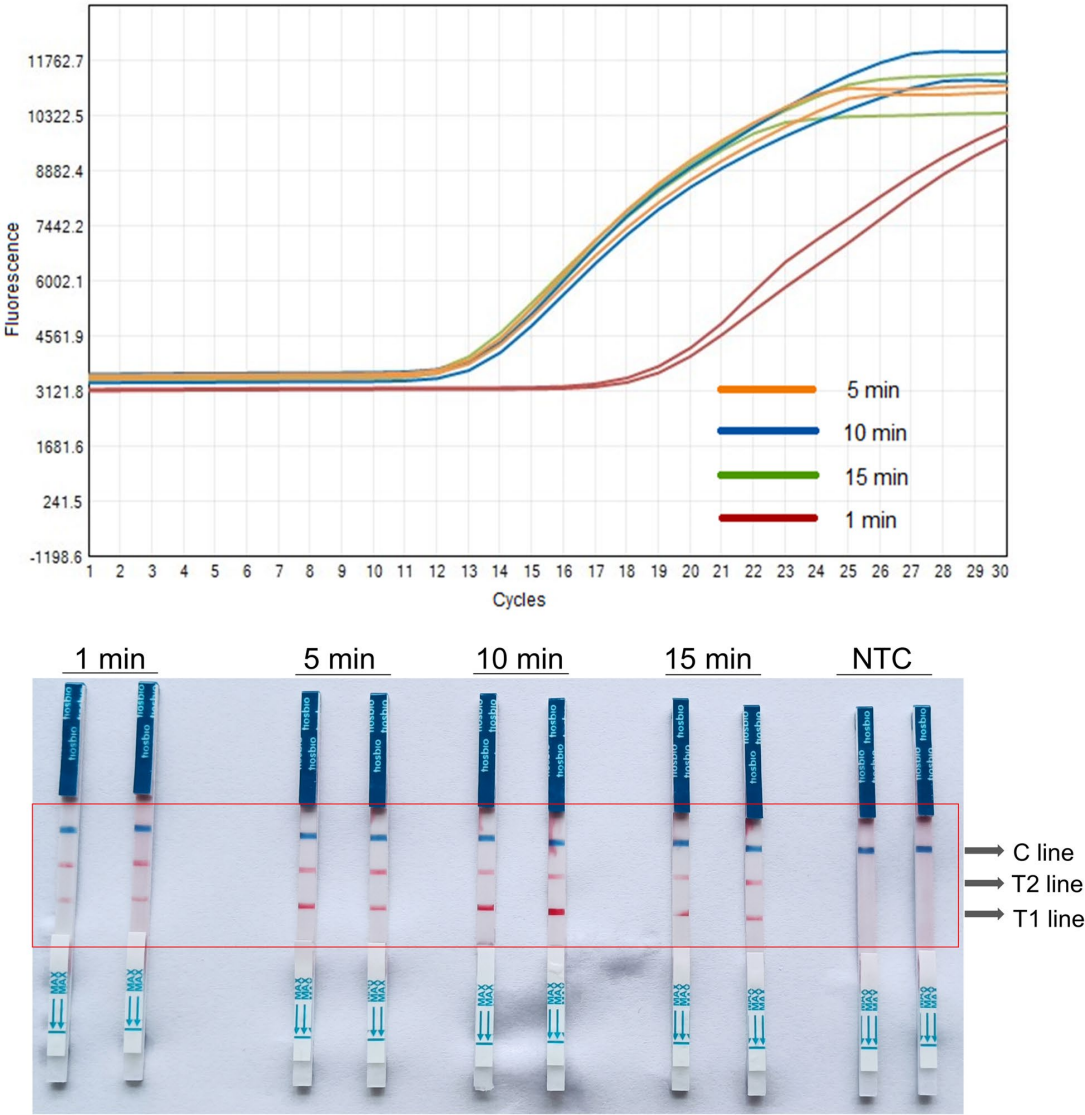
The RT-LAMP results from different lysis times. The fluorescence amplification curves were shown on the top, and the coloration reaction results were shown on the bottom. NTC, negative control. T1 line, Test line 1 (ACTB gene); T1 line, Test line 2 (N gene); C line, Control line

To optimize reaction conditions, we have further refined the reaction system. We first tested the reaction efficiency of original samples lysed by lysis buffer in comparison to nucleic acid samples extracted by kit. It was found that the amplification results of samples lysed directly with lysis buffer (CT value of 12) and those with nucleic acid (CT value of 11) demonstrated little variation (Fig. 4), indicating that the lysis buffer did not affect the efficiency of the entire reaction system. Furthermore, to identify the optimal lysis time, we experimented with lysis times of 1, 5, 10 and 15 minutes. Observations indicated that the lysate could initiate nucleic acid release roughly within 5 minutes (Fig. 5). Thus, the optimal lysis time determined in this study, was 5 minutes.

**Fig. 6:**
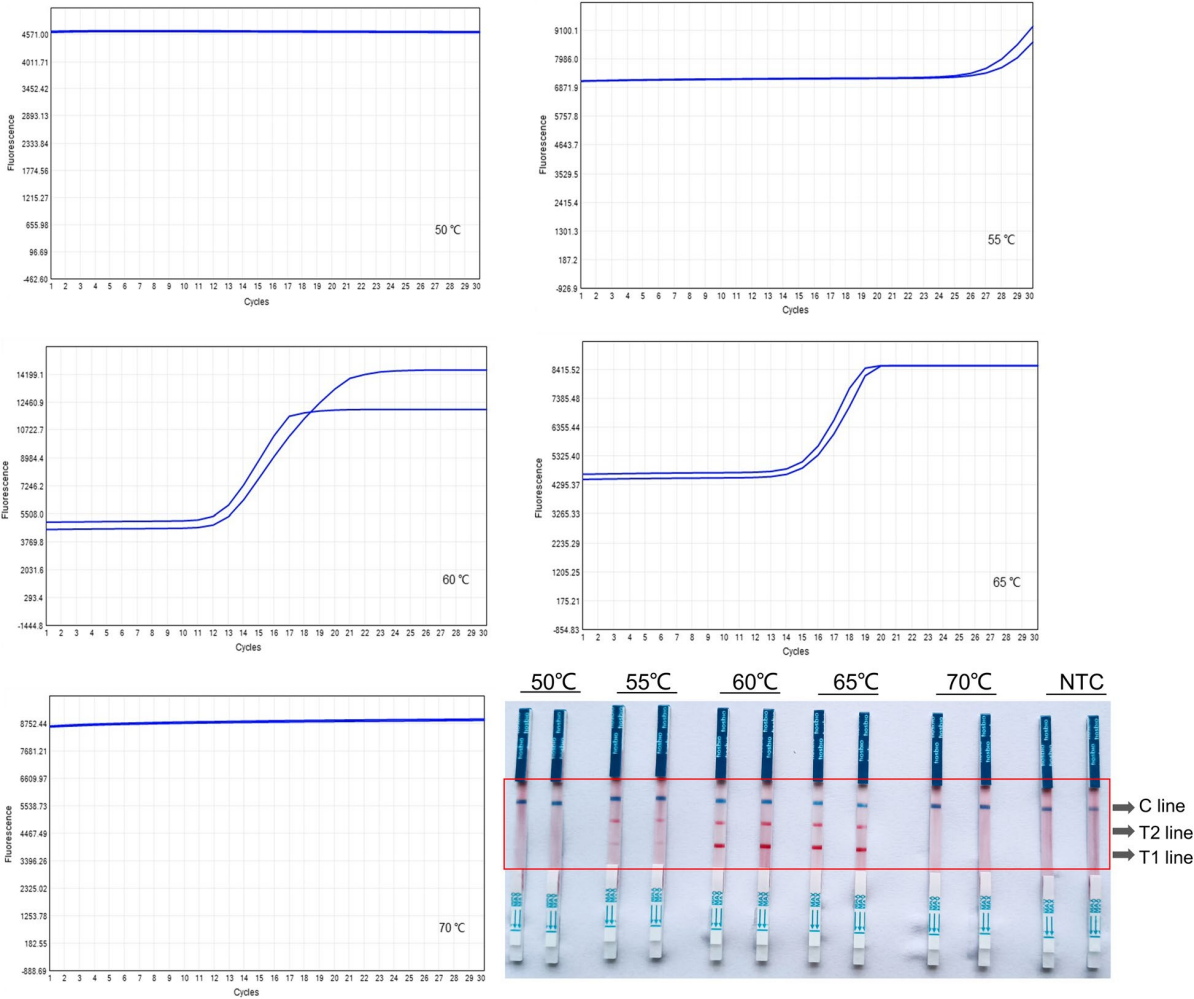
The results of RT-LAMP at different reaction temperatures. The fluorescence amplification curves were displayed on the top, and the coloration reaction results were shown on the bottom. NTC, negative control. T1 line, Test line 1 (ACTB gene); T1 line, Test line 2 (N gene); C line, Control line

Subsequently, the efficacy of RT-LAMP amplification was analyzed, varying the reaction temperatures and reaction times, while maintaining consistent template concentration. Experimental data showed that effective target amplification could occur between 55℃ and 65℃, though the peak efficiency was observed at 60℃ (Fig. 6). Therefore, the optimal reaction temperature for this assay was determined to be 60℃.

**Fig. 7:**
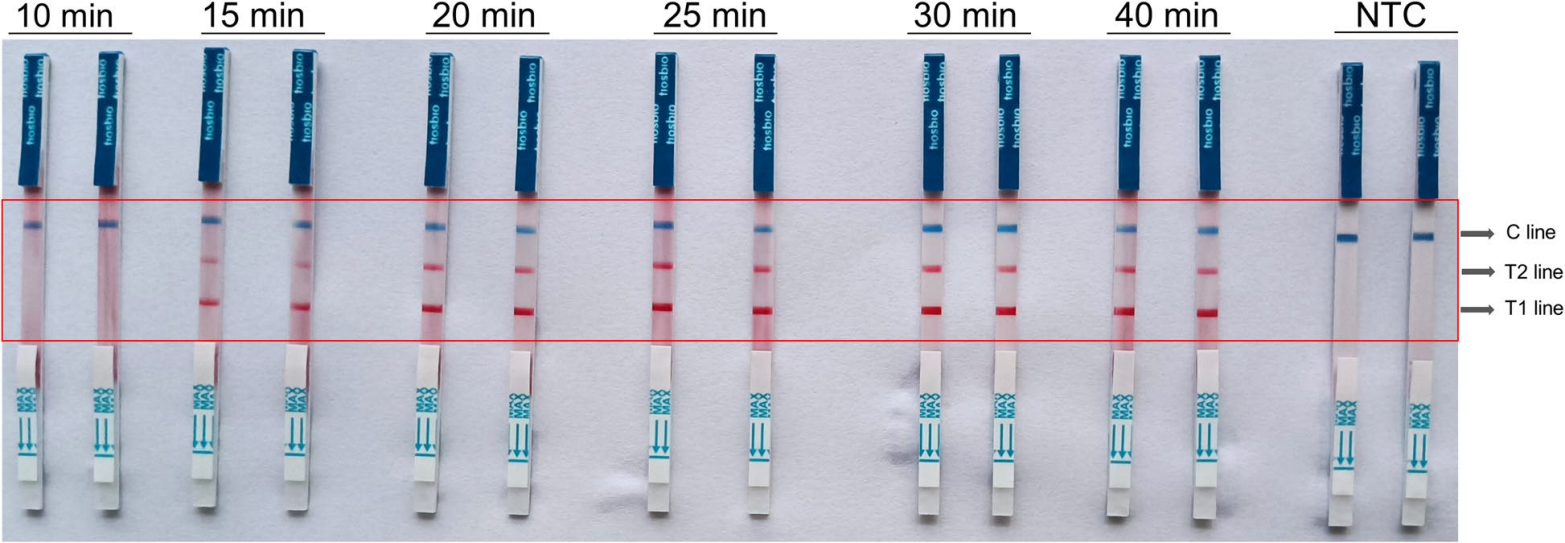
The coloration reaction results of RT-LAMP experiment with different reaction times. NTC, negative control. T1 line, Test line 1 (ACTB gene); T1 line, Test line 2 (N gene); C line, Control line

The reaction time results indicated that the reaction products could be interpreted using test strips after 15 minutes of reaction, with no discernable difference observed post-20 minutes (Fig. 7). However, to achieve the best detection effect, particularly of samples with low concentrations, the amplification time should be increased. Consequently, the whole reaction time was determined to be 30 minutes.

### Analyses of sensitivity and specificity

**Fig. 8:**
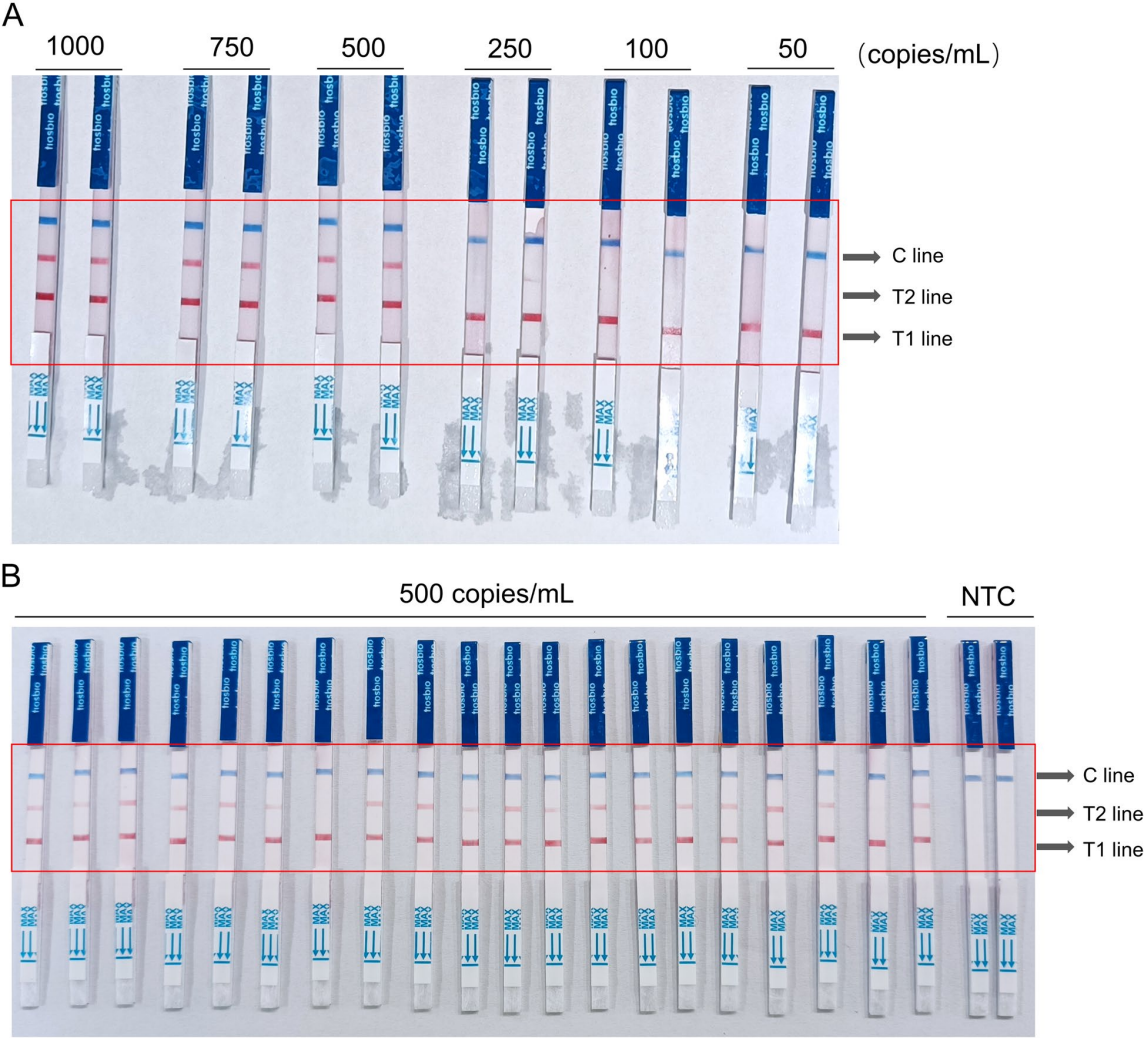
The level of detection (LOD) obtained using RT-LAMP assays. **A** The coloration reaction results of RT-LAMP assays for pseudovirus samples at different concentrations (50, 100, 250, 500, 750, and 1000 copies/mL); **B** The coloration reaction results of 20 repeated RT-LAMP assays for 500 copies/mL pseudovirus samples. T1 line, Test line 1 (ACTB gene); T1 line, Test line 2 (N gene); C line, Control line

Commercial SARS-COV-2-N pseudoviruses were used in a gradient dilution of 50, 100, 250, 500, 750, and 1000 copies/mL to identify the limit of detection (LOD) for RT-LAMP. Based on the results depicted in Fig. 8, the LOD for RT-LAMP was established at 500 copies/mL.

**Fig. 9:**
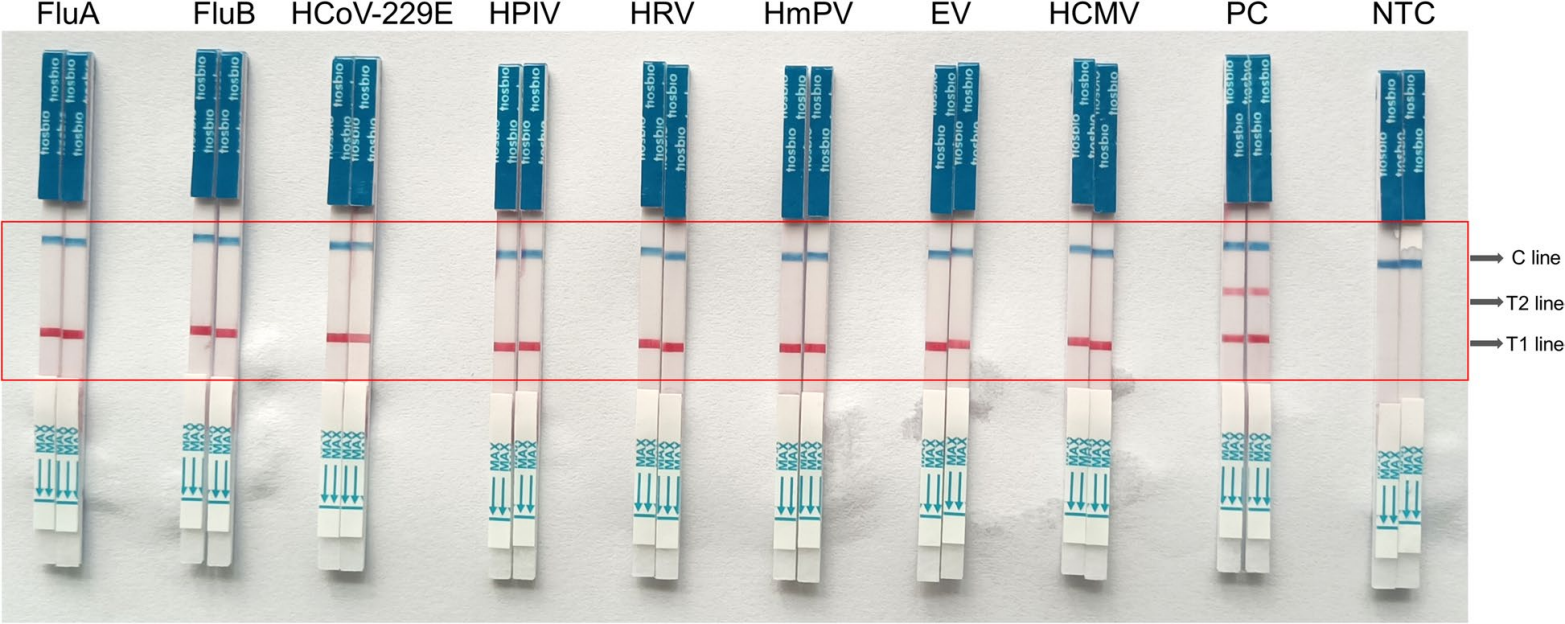
The coloration reaction results of RT-LAMP specificity test. FluA, influenza A virus; FluB, influenza B virus; HCoV-229E, human coronavirus 229E; HPIV, human parainfluenza virus; HRV, human rhinovirus; HmPV, human metapneumovirus; EV, enterovirus; HCMV, human cytomegalovirus; PC, positive control; NTC, negative control. T1 line, Test line 1 (ACTB gene); T1 line, Test line 2 (N gene); C line, Control line

The specificity results (Fig. 9) showed that the developed RT-LAMP assay in this study was unambiguously specific (100%) for the novel coronavirus, and had no cross-reactivity with either other human pathogenic coronaviruses or common viral pathogens - all tested negative.

### RT-LAMP tests of clinical specimens

Of the 498 clinical samples, 12 were disqualified (invalid qPCR or RT-LAMP tests), culminating in an evaluated sample size of 486 for determining the clinical application efficiency of RT-LAMP detection. Raw data are shown in Additional file 1. The results of RT-LAMP were benchmarked against the RT-qPCR test results (Table 2). The performance of RT-LAMP detection were: sensitivity-87.1%; specificity- 100%; positive predictive value-100%; negative predictive value-89.4%. The consistency between RT-LAMP and RT-qPCR assay results was high, at 93.8%, and Cohen's kappa was 0.813, within confidence intervals (0.635, 0.991). Further analysis of the CT values of false negative samples showed that the majority of these samples had CT values detected by RT-qPCR distributed between 28-30 (25/30, 83.33%), indicating that this range would make the interpretation of RT-LAMP results challenging. In other words, when the CT values of FAM and VIC channels were greater than 28, the RT-LAMP method had a lower detection rate in positive samples. However, in positive samples, the highest Ct value of FAM channel detected by RT-LAMP was 30.91, and the highest CT value of VIC channel was 31.26, both exceeding the positive judgment value of RT-qPCR detection. This indicated that the RT-LAMP method established in our study could complete the detection and screening of the novel coronavirus same as the commercial qPCR kit.

**Table 2:** The RT-LAMP results compared with that of qPCR

		RT-LAMP results		Total
		Negative	Positive	
qPCR results	Negative	254	0	254
	Positive	30	202	232
Total		284	202	486

## Discussion

A prompt and trustworthy diagnosis of the novel coronavirus is critical to constrain its widespread propagation. In this investigation, we have developed a swift and straightforward RT-LAMP assay for the detection of the novel coronavirus. This methodology, which uses test strips for chromogenic interpretation, permits completion of the whole procedure in under 30 minutes and is user-friendly.

At present, detection methods for novel coronavirus include RT-qPCR, Next-Generation Sequencing (NGS), immunological tests for antigens and antibodies, etc. As the gold standard of the detection of SARS-CoV-2, RT-qPCR can accurately detect COVID-19 through standardized laboratory testing operations [13, 14]. Due to the restrictions in laboratory-based molecular detection’s capacity and its extensive turnaround time, the screening of a large number of people demands abundant resources, including manpower, material resources, and highly trained laboratory professionas. Therefore, for large-scale population screening that are beyond regular laboratory conditions, alternative detection methods like RT-LAMP, real-time technologies, and isothermal amplification technologies are recommended. These methods prove efficient for screening SARS-CoV-2 infection [15, 16]. While the NGS technology can analyze the SARS-CoV-2 genome, it is costly and time-consuming, rendering it unsuitable for mass screenings, and it is predominantly used in the genome analysis and virus mutation analysis [17]. Although antigen-antibody immunoassay is straightforward and speedy in operation, the time window for detection of SARS-CoV-2 infection by this assay is very narrow, and can suffer from low sensitivity and specificity [18, 19].

Several LAMP methods for SARS-CoV-2 have been developed leveraging rapid colorimetric detection of target genes. Nevertheless, a significant drawback is the inability to execute multiple tests concurrently. If multiple target tests are deemed necessary, each target gene must undergo individual testing [11]. Because, the single-tube multiplex LAMP detection will increase the number of times the lid is opened during the experiment, this will greatly increase the risk of aerosol contamination, resulting in false positives [10]. In the practice of large-scale clinical trials, individual detection of multiple target genes will multiply the number of tests, which diminishes the rapid diagnostic benefits offered by LAMP testing. Therefore, the RT-LAMP with lateral flow assay incorporating human-derived internal reference genes developed in this study offers more practical edge for clinical screening applications in terms of maximizing the assessment of sample quality for better interpretation.

The integration of RT-LAMP and LFA method in this study yielded a technique capable of executing the entire assay within 30 minutes, testing two target genes at a stable temperature of 60°C, demonstrating remarkable consistency compared to the fluorescent PCR nucleic acid assay. We described the accuracy of RT-LAMP detection method for SARS-CoV-2 through a comparative analysis with the results of fluorescence quantitative PCR, by determining the likelihood ratio. Congruence with RT-qPCR results stood at 93.8%. This index indicated that the RT-LAMP test, as developed in our study, has proven diagnostic value for SARS-CoV-2. Clinical trial results revealed that the RT-LAMP assay was capable of attaining detection levels at 500 copies/mL, which could meet the screening of patients infected with SARS-CoV-2. Further analysis of the clinical sample results revealed that RT-LAMP demonstrated false negatives compared to qPCR results when the CT value surpassed 28, essentially coinciding with the lower detection limit for qPCR. In addition, compared to the qPCR method, the RT-LAMP method significantly reduces the turnaround time and the expertise requirement for personnel. Our assay also displayed advantages in timeliness and simplicity, and did not require separate steps for reverse transcription and amplification [6]. Notably, the results of our method are decipherable by unaided visual inspection, eradicating the prerequisite for specialized equipment. Finally, the RT-LAMP method in this research showed no cross-reactivity with other viruses known to cause similar respiratory diseases and demonstrated a distinctive 100% specificity for SARS-CoV-2.

## Conclusions

The robust RT-LAMP assay formalized in this study can be a supplementary method for monitoring vast numbers of exposed individuals, thereby boosting the proficiency of screening procedures in healthcare centers and the public arenas. It is particularly advantageous in areas with limited laboratory resources since the RT-LAMP assay can bypass RNA extraction and require no specialized testing instruments.

Subsequent to this study, further optimization of the RT-LAMP assay has been achieved through the creation of lyophilized reagents for reactions. These can be stored and transported at ambient temperature. For this simplified single-tube RT-LAMP detection method, if the execution time can be shortened and sensitivity can be increased in future research, it will become a more promising SARS-CoV-2 POCT product.

### Supplementary Information

**Additional file 1.** 

## Data Availability

The datasets used during the current study are available in NCBI (GenBank accession no.: NC_045512, location 28, 274-29, 533).
